# Organizational Principles of Biological Systems

**DOI:** 10.3390/biology15060500

**Published:** 2026-03-20

**Authors:** Roberto Carlos Navarro-Quiroz, Kelvin Navarro Quiroz, Victor Navarro Quiroz, Antonio Gabucio, Ricardo Fernández-Cisnal, Noelia Geribaldi-Doldán, Cecilia Fernandez-Ponce, Ismael Sánchez Gomar, Yesit Bello Lemus, Eloina Zárate Peñata, Lisandro A. Pacheco-Lugo, Leonardo C. Londoño-Pacheco, Martha Rebolledo Cobos, Antonio Acosta Hoyos, Diana Pava Garzon, José Luis Villarreal Camacho, Elkin Navarro Quiroz

**Affiliations:** 1 Center for Research in Critical Dynamics, Barranquilla 080002, Colombia; robertcnavarro@gmail.com (R.C.N.-Q.);; 2 Instituto de Química, Universidade Estadual Paulista (UNESP), Câmpus de Araraquara, Araraquara 14800-900, Brazil; 3 Tecnológico de Antioquia Institución Universitaria, Medellín 050036, Colombia; 4 Instituto de Investigación e Innovación Biomédica de Cádiz (INiBICA), 11009 Cádiz, Spain; 5 Departamento de Biomedicina, Biotecnología y Salud Pública, Facultad de Medicina, Universidad de Cádiz, 11003 Cádiz, Spain; 6 Centro de Investigaciones en Ciencias de la Vida (CICV), Facultad de Ciencias Básicas y Biomédicas, Universidad Simón Bolívar, Barranquilla 080002, Colombia; yesit.bello@unisimon.edu.co (Y.B.L.); antonio.acosta@unisimon.edu.co (A.A.H.); 7 Facultad de Ciencias de la Salud, Fundación Universitaria San Martin, Puerto Colombia 081007, Colombia; 8 Instituto Nacional de Salud de Colombia, Bogotá 111321, Colombia; 9 Facultad de Salud, Universidad Metropolitana de Barranquilla, Barranquilla 080001, Colombia

**Keywords:** physics of life, complex systems, self-organization, emergence, information theory, biological organization, criticality

## Abstract

How does the complex, adaptive, and autonomous organization of life emerge from the laws of physics and information? This review argues that the answer lies in a convergent set of universal organizational principles that constitute a physical and informational grammar of the living. Living systems are dissipative structures that achieve organizational closure—materially and energetically open, yet causally closed—thereby attaining genuine autonomy and agency. Their architecture exhibits fractal and modular scaling laws that maximize energy flow, robustness, and evolvability under universal physical constraints. Critically, organisms operate at critical transitions—zones of controlled instability where fluctuations amplify information processing, transforming noise into adaptive signal. This self-organized criticality enables functional degeneracy, relational redundancy, and evolutionary antifragility. Cognition emerges as a distributed process of active inference, operating through a predictive–corrective cycle that integrates perception, action, and learning under the Free Energy Principle. From molecular networks to ecosystems, the same physico-informational grammars unfold recursively, revealing a deep organizational holography: the principles of organization are replicated across scales. Evolution under the Law of Increasing Functional Information is not random drift, but a directional expansion of functional complexity—a thermodynamic gradient towards greater agency. This synthesis challenges biological exceptionalism: the trajectory from thermodynamics to cognition is continuous, physically constrained, and potentially inevitable. Life does not violate physical laws—it fulfills them in regimes of high informational complexity, instantiating fundamental principles in self-organized architectures capable of prediction, memory, and purpose. The objective of this work is to articulate how the synthesis of these principles not only unifies physics and biology, but also illuminates the profound continuity between thermodynamics, chemistry, informational constraints, organization, and the mind.

## Simple Summary

Living systems instantiate a universal physical–informational–biological grammar—a convergent set of organizational principles that explain how structure, function, and cognition emerge from thermodynamic, chemical, and informational laws, incorporating inherited genetic information expressed through peptides and folded functional proteins. Organisms are dissipative structures achieving organizational closure: materially open but causally self-contained, actively producing the components that define their identity. This autonomy manifests through fractal-modular architectures optimizing energy flow under universal constraints, and through operation at self-organized criticality—dynamic regimes maximizing information processing at the edge of chaos. These principles generate functional degeneracy and antifragility, transforming environmental perturbations into evolutionary innovation. Cognition emerges not as neural exclusivity but as distributed active inference: predictive systems minimizing surprise across all biological scales. Evolution follows the Law of Increasing Functional Information—directional expansion toward greater functional complexity, not random drift. This synthesis challenges biological exceptionalism: life does not violate physical laws but fulfills them in high complexity regimes, revealing organizational holography where principles replicate fractally from molecules to ecosystems. We establish falsifiable predictions through metabolic scaling, critical avalanche distributions, and paleogenomic information density. Applications span precision medicine (disease as phase transitions from criticality), synthetic biology (engineering autopoietic systems), and biomimetic AI (organizational intelligence beyond computation).

## 1. Introduction

The Essence of Universal Principles of Biological Organization Life is distinguished from inert matter by its capacity to maintain organization, adaptability, and autonomy in an environment subject to fluctuations and increasing entropy. This organization is not arbitrary; it responds to universal principles that govern the emergence, stability, and evolution of complex systems far from global equilibrium. Understanding these principles is the central goal of a Physics of Life, in which biology is interpreted as a particular manifestation of the laws governing the self-organization of matter, energy, and information [1,2]. Unlike closed systems that tend toward disorder (increasing entropy), organisms are open entities that constantly exchange energy, information, and matter with their surroundings. This exchange allows them to export entropy and maintain an internal state of high organization and low entropy, an indispensable condition for life (dissipative structures) [3,4,5]. The continuous flow of energy not only sustains this order but also drives the system far from thermodynamic equilibrium, where non-linear dynamics arise. Under these critical conditions, small internal fluctuations can be amplified, generating instabilities that culminate in the spontaneous breaking of symmetries. This process is the engine that originates complex patterns and new levels of spatiotemporal organization, a funda- mental mechanism explained by the theories of Prigogine [3] and Turing [4]. Consequently, biological stability should not be viewed as static homeostasis—the mere maintenance of constancy—but rather as homeodynamics: a dynamic equilibrium that preserves organizational integrity through continuous change. Life, therefore, is not a fixed state of being, but a continuous process of becoming.

Life is maintained not despite disequilibrium, but because of it [4]. However, the key distinguishing feature of living systems resides not solely in their dissipative structure, but in their organizational closure: the capacity to actively produce and maintain their own components [6,7,8]. This principle, formalized by Maturana and Varela’s [6] autopoiesis, Rosen’s [7] (M,R) systems, and Kauffman’s [9] autocatalytic sets, defines biological autonomy. A system that is causally closed but materially open is capable of sustaining its identity and purpose, generating an internal normativity: action oriented toward its own viability [8]. Biological organization is further expressed in fractal and modular architectures, where self-similarity and hierarchy optimize energy and information exchanges [9]. Models such as that of West, Brown, and Enquist (WBE) demonstrate that fractality constitutes an optimal physical solution for transport efficiency and functional robustness [10,11]. Modularity, in turn, favors evolvability and the capacity for structural adaptation to perturbations [10]. At a dynamic level, living systems operate near self-organized criticality—the “edge of chaos”—a regime that maximizes information processing and flexible response capacity in changing environments [8,9,10,11,12,13,14]. This balance between order and fluctuation is a universal property of biological complex systems: from neural networks to ecosystems, criticality confers adaptability, memory, and functional creativity [15,16,17,18]. Adaptive behavior is sustained by functional degeneracy—the existence of multiple structural configurations capable of performing the same function—and by antifragility, the ability to improve in the face of perturbations [18,19]. These properties make life a system that learns from disorder and transforms variability into a source of innovation [20]. Evolution can be understood as a process directed by physical–informational constraints. The Law of Increasing Functional Information (LIFI) posits that evolutionary systems tend toward an increase in functional information—the capacity to sustain and execute complex functions—which introduces a natural directionality into biological evolution [20]. This review addresses a fundamental question: how does the complex, adaptive, and autonomous organization of life emerge from the laws of physics and information? We argue that the answer lies not in the molecular components, but in a convergent set of organizational principles. These principles—dissipation, organizational closure, fractality, modularity, criticality, degeneracy, antifragility, and increasing functional information—constitute a universal grammar of life. Through them, living matter is revealed as a physical–informational system capable of generating order, meaning, and cognition from the continuous flow of energy and data. The objective of this work is, therefore, to articulate how the synthesis of these principles not only unifies physics and biology but also illuminates the profound continuity between thermodynamics, organization, and the mind.

## 2. Results

### 2.1. State of the Art and Main Formal Models

Understanding the universal organizational principles that sustain life requires integrating three conceptual domains: non-equilibrium thermodynamics, the theory of self-organization, and the logic of organizational closure. Together, these approaches explain how living systems emerge, maintain themselves, and evolve as coherent structures far from global equilibrium, transforming energy and information to sustain their functional identity [21]. Specifically, the thermodynamic domain holds that the Second Law of Thermodynamics acts as a fundamental constraint that drives the emergence of structure in dissipative systems. This driving force is manifested so that processes emerge to reduce the applied gradient, a phenomenon which, if dynamic conditions permit, is recognized as self-organization. Consequently, the expected outcomes of this thermodynamic mandate are that as biosystems grow and develop, they must increase their total dissipation, leading to greater control of complexity, energy flow, diversity, and hierarchical levels [21]. Furthermore, this thermodynamic imperative provides the critical lens for interpreting biological success and failure, which directly supports the conceptual domain of organizational closure mentioned in the paragraph. The summary’s corollary—that organisms which do not increase total dissipation are essentially organisms “dedicated to death” (like during aging)—and its observation that surviving species are those that funnel energy into reproduction and contribute to autocatalytic processes that increase the ecosystem’s total dissipation, provides the practical, observable evidence for a system maintaining its “functional identity” far from global equilibrium. In essence, the opening paragraph lays out the required intellectual toolkit, while the summary translates that toolkit into a set of universal organizational principles that govern the growth, survival, aging, and complexity of all biosystems [21].

### 2.2. Thermodynamic Foundation: Dissipation, Instability, and Organization

Living organisms are thermodynamically open systems that maintain their state of low entropy (high order) thanks to a continuous flow of energy, information, and matter, exporting disorder to the environment. This principle, already stated by Schrödinger, constitutes the physical basis of life as a process of maintained negentropy [22].

The theory of dissipative structures developed by Ilya Prigogine and collaborators [3] demonstrated that, far from global equilibrium, non-linearity in system dynamics allows for the amplification of small fluctuations under conditions of energetic forcing, leading to the emergence of new spatiotemporal organizational states. This process can be represented as a fundamental causal sequence [23]:

Energy, material gradients, and information → dissipation → instability → symmetry breaking → emergent order.

This mechanism not only explains self-organization in physical systems but also biological morphogenesis, where Turing patterns emerge as a consequence of diffusive–reactional instabilities that break spatial symmetries and give rise to ordered structures.

From this perspective, biological stability does not correspond to a static homeostasis, but to a homeodynamics, in which equilibrium is redefined as a continuous oscillation around dynamic attractors. Thus, life is interpreted as a form of organized matter that is maintained “on the edge of instability,” using energy dissipation not as a cost, but as the engine of its own organization [24].

Non-equilibrium thermodynamics therefore provides the physical framework that explains how living systems can maintain order through the flow of entropy. Self-organization emerges as a natural consequence of the critical conditions imposed by functional information and energy gradients, establishing the material bases for the emergence of functional hierarchies, metabolic networks, and biological rhythms.

Beyond linear energy gradients, the formation of cyclic processes—such as metabolic loops, information processing, ecological recycling, and cellular oscillations—is essential for sustaining biological organization. As noted by [25,26], cycles enable the storage and coherent redistribution of energy, facilitating self-maintenance and increasing functional coherence. In living systems, cycles are inseparable from compartmentalization and modularity, reflecting a deeper principle of dynamical closure and coherence.

### 2.3. The Physical Engine of Autonomy: Circular Thermodynamics and QED Coherence

While dissipative structures explain the emergence of order, the maintenance of biological identity requires a mechanism that goes beyond simple energy flux. As pointed out by Morowitz and Ho [25], the essential feature of life is not merely the flow of energy, but the formation of cycles. In fluid dynamics, even elementary dissipative structures like Bénard–Raleigh cells rely on the liquid’s capacity to store thermal energy and cycle it through density changes, rather than on the external gradient alone. In biology, this principle of “Circular Thermodynamics” implies that organisms are capable of detaining energy within internal loops, creating a delay between input and output that constitutes the physical basis of agency [27,28].

However, the question remains: what physical mechanism guarantees the synchronization required for these cycles and for the modular compartmentalization of life? The answer lies in the quantum electrodynamic (QED) coherence of biological matter, particularly the “water connectome” [29].

Biological water does not behave as a bulk liquid but exists in a dual phase, including a coherent fraction where molecules oscillate in phase with an electromagnetic field. This coherent ground state is associated with an energy gap, which protects the system against thermal noise. This gap renders the coherent state thermodynamically desirable, naturally driving the system toward self-preservation (homeodynamics) and providing a physical derivation for teleonomy. Furthermore, this coherence is the “an inescapable, physically grounded, and increasingly well-supported condition” for the emergence of the organizational principles discussed in this review:

Modularity and Closure: Coherent domains provide dynamic compartmentalization without rigid walls, allowing for the distinct yet thermodynamically coupled processes required for organizational closure.

Fractality: There is a mathematical isomorphism between coherent quantum states and self-similar (fractal) structures; where fractality is observed, a coherent dynamic is in force.

### 2.4. Logic of Self-Fabrication: Organizational Closure and Autonomy

What distinguishes living systems from other dissipative structures is not just their ability to sustain order, but their organizational closure. From this perspective, biological information acquires causal efficacy only insofar as it is embedded in the physical configuration of the system itself, reinforcing the principle that in living systems structure is functional [30]: the property of actively producing, repairing, and maintaining the components that constitute them. This principle defines biological autonomy, understood as a system’s ability to regenerate the processes that make its own existence possible [31].

Three convergent formalisms have modeled this property with remarkable theoretical consistency:

(a) Autopoiesis (Maturana and Varela) [6]: Living beings are networks of production processes that continuously regenerate the components that constitute the network itself and its boundary, thereby defining an autonomous operational identity. This approach emphasizes that organization, not material composition, is what distinguishes the living [12,32].
(b) (M,R) Metabolism-Repair Systems (Rosen) [7]: Life is characterized as a system “closed to efficient causality,” where each functional component is, in turn, a product of another component of the system. This causal closure implies an essential noncomputability, which differentiates organisms from machines, as an organism cannot be fully described or predicted from an external algorithmic description.
(c) Autocatalytic Sets (Kauffman) [9]: Beyond a critical threshold of complexity, networks of chemical reactions can become catalytically closed and self-sustaining. This model offers a plausible formalization for the emergence of coherent metabolism and the initial molecular self-organization of life [33].

In all these frameworks, biological agency, the ability to act with purpose, emerges as a thermodynamic consequence of organizational closure. At the cellular level, this closure is physically instantiated through thermodynamically coupled processes, such as ATP-driven primary active transport across anisotropic membranes. A system that must maintain itself under conditions of viability necessarily acts to preserve its internal structure, generating self-referential behaviors oriented toward its own persistence [34].

Organizational closure, therefore, explains not only autonomy but also the teleonomy inherent in living systems: their tendency to preserve identity through processes of self-fabrication and self-regulation. From a physical–informational viewpoint, these systems can be conceived as inferential entities that process information to maintain their internal coherence against external entropy, which anticipates the later articulation with the principles of active inference and free energy.

The emergence of organizational closure and modularity is physically underpinned by quantum electrodynamic coherence, particularly within the aqueous matrices of living systems [35]. This coherence facilitates long-range coordination, efficient energy transfer, and fractal self-similarity, enabling biological matter to operate as a unified, yet dynamically open, phase of matter.

### 2.5. Universal Organizational Principles

Life can be understood as a particular expression of organizational principles that govern matter far from global equilibrium. These principles of autopoiesis, fractality, modularity, criticality, distributed cognition, antifragility, and the increase in functional information act as organizational invariants that cut across biological scales, from molecular networks to cognitive systems and ecosystems [36]. Together, they constitute a physical and informational grammar of the living, where structure and dynamics are inseparable.

### 2.6. Autopoiesis, (M,R) Systems, and Autocatalytic Sets

An autopoietic system actively maintains its identity through the continuous production and repair of its own components. In the framework proposed by Maturana and Varela [6], living beings are defined as networks of production processes that regenerate both the network itself and its boundary, establishing a distinct internal operational identity [7]. Rosen formalized this idea in (M,R) systems, showing that organisms are “closed to efficient causality,” meaning that each function within the system is a product of another internal function, generating a causal circularity not reducible to external mechanisms [8]. Kauffman, for his part, demonstrated that when chemical reaction networks reach a critical density, closed and self-sustaining autocatalytic sets emerge, capable of maintaining a coherent metabolism [6]. These models converge on a fundamental thesis: life is self-referential, a system that produces the conditions of its own persistence. Causal closure implies internal normativity: the system acts to keep itself within a domain of viable states, giving rise to an immanent form of biological purpose.

### 2.7. Fractality and Modularity

Biological organization is not random: it follows hierarchical, modular, and fractal architectures that reflect universal principles of optimization under physical constraints. Modularity implies that systems are composed of subsystems that are densely interconnected internally but weakly coupled to each other. This pattern confers local robustness against perturbations and global evolvability, by allowing the functional recombination of “building blocks” [9]. Fractality, on the other hand, reflects structural self-similarity across scales. It constitutes an optimal solution for maximizing exchange surface area within a limited volume, as occurs in the lungs, vascular systems, or neural networks [13]. The West, Brown, and Enquist (WBE) model formalized this principle, showing that allometric laws—such as the metabolic rate M*—emerge from the fractal optimization of energy flow [10]. Modularity and fractality, together, define the universal architecture of living networks: hierarchical structures that balance efficiency, resilience, and the capacity for innovation.

Fractal architectures in biology are not only geometric optimizations but also reflect underlying coherent quantum states, where self-similarity emerges from resonant energy dynamics across scales [35]. This coherence supports both structural integrity and functional adaptability, linking physical symmetry breaking to biological organization.

### 2.8. Criticality

Numerous biological systems—from gene regulation to cortical activity—operate near a critical point, at the “edge of chaos.” Self-organized criticality (SOC) posits that complex systems can spontaneously evolve toward this intermediate state, where stability and variability coexist [10,11]. In this regime, life exploits the statistical properties of phase transitions to optimize its performance:

▪ It maximizes the dynamic range and sensitivity to stimuli.
▪ It optimizes information processing and transmission.
▪ It maintains a balance between robustness and adaptive flexibility.

Evidence of critical dynamics has been observed in neural avalanches that follow power laws, in self-regulated gene networks, and in ecosystems showing scaling of population fluctuations [14,37]. Criticality, in this context, is not an evolutionary accident but a boundary condition where information and energy are optimally coupled, allowing the system to respond creatively to perturbations without losing coherence.

### 2.9. Distributed Cognition

The functional coherence of living systems emerges from distributed self-organization rather than centralized control. This principle is manifest from insect colonies and immune systems to the brain. Models like Kuramoto’s [16] describe the spontaneous synchronization of coupled oscillators, a universal mechanism by which systems achieve temporal coherence from local interactions [15]. On a more abstract level, Friston’s [17] Free Energy Principle (FEP) formalizes cognition as active inference: living systems maintain an internal model of the world and act to minimize “surprise” or prediction error, unifying perception, action, and learning under a Bayesian principle of self-evidence [16]. Thus, cognition ceases to be an exclusive attribute of the brain and is redefined as an extended and multi-scalar property: the continuous process by which life models, anticipates, and regulates its states in an uncertain environment.

### 2.10. Antifragility and Degeneracy

Living systems do not just resist disorder: they benefit from it. Antifragility, as stated by Taleb [18], describes systems whose structure improves in the face of perturbations, volatility, or stress. Its biological foundation lies in functional degeneracy, the existence of multiple structurally different components capable of fulfilling the same function [18]. This diverse redundancy allows for the absorption of variations without loss of performance and enables evolutionary innovation: new functions can emerge without sacrificing existing ones. Degeneracy, therefore, constitutes the structural substrate of antifragility, while criticality acts as its dynamic condition. Together, these properties make life a robust, evolutionary, and creative system that transforms uncertainty into a source of adaptation.

### 2.11. Law of Increasing Functional Information (LIFI)

Biological evolution, far from being a random drift, shows directional trends in the accumulation of organized complexity. The Law of Increasing Functional Information (LIFI) proposes that functional information—understood as a system’s ability to maintain its integrity and execute functions—tends to increase in open systems subjected to selection for function [38]. This law is analogous to the Second Law of Thermodynamics but oriented toward information: while entropy measures energy disorder, functional information measures operational order [36,39]. Throughout evolution, systems tend to explore configurations that increase their ability to process energy and information more efficiently. The LIFI offers a quantitative formulation of evolutionary directionality: life progresses toward states of greater integration, coordination, and informational processing, which can be observed in the expansion of metabolic networks, cellular specialization, and the emergence of cognition [19].

### 2.12. Conceptual Synthesis and Structural Analogies: The Holographic View

The principles described—dissipation, organizational closure, fractality, modularity, criticality, degeneracy, antifragility, and increasing functional information—do not act in isolation. They manifest in a self-similar and recursive manner at all levels of biological organization, from macromolecules to the biosphere. This holographic convergence suggests that each level of organization reflects the logic of the others: the same patterns of self-organization, distributed control, and energy optimization are repeated, adjusting to the specific constraints of each scale.

### 2.13. The Immune System: Autonomy, Fractality, and Distributed Cognition

The immune system is a paradigmatic expression of this holographic view. It is, first and foremost, a dissipative structure that maintains the organizational closure of the “self” through the continuous production of cells, receptors, and signals that sustain immunological identity [40]. Its fractal and modular architecture—from lymph nodes to antigenic recognition microdomains—optimizes the transport of information and cells throughout the body, maximizing efficiency under energy constraints. The immune system operates near a dynamic criticality, where small stimuli can be amplified into coordinated responses without loss of stability, analogous to the “neural avalanches” observed in the brain [41]. This condition allows it to respond to an unpredictable universe of pathogens without collapsing into systemic inflammation. Its antifragility is manifested in immunological memory: exposure to perturbations (pathogens or antigens) strengthens the network, expanding its functional repertoire. Immunological degeneracy—multiple clones, pathways, and molecular redundancies that converge on equivalent functions—confers resilience against mutations or antigenic evasion [42]. Finally, the immune system can be understood as a distributed cognitive system: it learns, remembers, and makes decisions without a control center, operating under a principle of active inference that minimizes surprise in the face of the pathogenic environment, as formalized by the Free Energy Principle [16]. Its dynamics thus conform to the same principles that govern the brain, but in a different topology: that of the extended body.

### 2.14. The Brain: Criticality, Modularity, and Informational Efficiency

The brain is another instance where holographic self-organization becomes evident. Neural networks exhibit hierarchical modularity and fractal geometry, optimizing signal transmission under metabolic constraints, analogously to vascular or lymphatic networks [43]. Experimental evidence indicates that cortical activity operates near a critical point, where neural connectivity oscillates between order and chaos, maximizing computational capacity and adaptive plasticity [44]. The brain maintains functional closure in its dynamics of synaptic self-fabrication, generating and reinforcing connections that preserve the internal coherence of the system, a neurobiological form of the principle of autopoiesis. In terms of the Free Energy Principle [16], the brain acts as an active inference system that minimizes the discrepancy between its internal predictions and sensory reality, i.e., an information processor that maintains its viability by reducing uncertainty.

### 2.15. The Microbiome: Critical Ecological Network and Co-Autonomy

The microbiome represents an intermediate scale between the cellular and the ecological, where the principles of modularity, degeneracy, and antifragility are clearly expressed. The microbial community constitutes a distributed and redundant metabolic network: different taxa can perform similar functions (functional degeneracy), ensuring stability in the face of environmental changes [45]. These networks exhibit critical properties, adapting rapidly to perturbations through phase transitions that reorganize the relative abundance of species without collapsing the ecosystem’s functionality. Their ecological fractality is reflected in the self-similarity of connectivity and co-occurrence patterns at different scales, from intestinal microhabitats to global communities, a trait shared with intracellular metabolic networks [46]. The microbiome, moreover, participates in the extended cognition of the organism: it modulates behavior, the immune system, and neuroendocrine homeodynamics. In this sense, the biological individual cannot be separated from its symbiotic ecosystem: autonomy is always co-autonomy [47].

The microbiome operates through tightly coupled metabolic cycles that enable energy storage and redistribution, a principle highlighted by [27] in her reformulation of the Second Law for living systems. These cycles, sustained by electrodynamic coherence, allow for rapid adaptation and minimal entropy production, even under perturbation.

### 2.16. Tissues: Self-Organization and Morphodynamic Patterns

In multicellular tissues, the principles of self-organization are expressed in morphogenesis and tissue homeodynamics. Turing models [4] and Prigogine’s [3] dissipative structures [48] explain how chemical gradients and local fluctuations are amplified to generate self-organized spatial patterns (e.g., skin pigmentation, glandular architecture). Cellular interactions exhibit critical feedbacks that maintain the balance between proliferation and differentiation, and the fractal geometry of the vascular system optimizes nutrient diffusion. As a whole, tissues operate as multi-scalar systems, where cellular organization reflects—in a reduced version—the dynamics of the entire organism [49].

### 2.17. Ecosystems and Biosphere: Critical Flow Networks and Resilience

At the macroscopic level, ecosystems also show the signature of universal organizational principles. Trophic networks and energy transport networks are modular and fractal, which allows for local stability and global flexibility in the face of perturbations. Population fluctuations follow power laws, indicative of self-organized critical dynamics, where ecosystems are maintained in dynamic equilibrium at the edge of instability [50]. Ecological antifragility is observed in adaptive succession: environmental perturbations not only select but also induce functional reorganization, increasing the informational complexity of the system. Thus, the entire biosphere can be understood as an autopoietic meta-system that maintains its organization through dissipative cycles, energy transfers, and informational couplings at multiple scales [51].

### 2.18. Organizational Holography: A Universal Grammar of Life at All Scales

Life obeys a universal organizational grammar. Across biological scales, dissipation maintains the distance from global equilibrium, organizational closure ensures autonomy, fractality and modularity optimize structural efficiency, criticality maximizes adaptability and information processing, degeneracy and antifragility confer evolutionary robustness, and increasing functional information processing orients evolution toward greater degrees of integration.

▪ Dissipation maintains the distance from global equilibrium.
▪ Organizational closure ensures autonomy.
▪ Fractality and modularity optimize structural efficiency.
▪ Criticality maximizes adaptability and information processing.
▪ Degeneracy and antifragility confer evolutionary robustness.
▪ Increasing functional information processing orients evolution toward greater degrees of integration.

This self-similar repetition of principles generates a holographic view of life, in which each level—molecular, cellular, organic, cognitive, and ecological—reflects the totality of the system. Biological organization is, thus, a fractal manifestation of the laws of informationally active matter, a continuity between thermodynamics, information, and cognition.

### 2.19. Falsifiable Predictions, Controversies, Limits, and Experimental Projections

Although the framework of a Physics of Life offers a unifying theoretical synthesis, its validity depends on its ability to generate testable predictions and to confront its own conceptual limits. The strength of the approach lies in its falsifiability: the possibility of subjecting its universal organizational principles—criticality, organizational closure, and the Law of Increasing Functional Information (LIFI)—to rigorous empirical tests that can distinguish between real universality and superficial analogy [52].

### 2.20. Controversies and Limits

Despite its integrative power, this framework faces three main objections:

(a) Context Dependency and Historical Contingency: The “small world” approach and the search for universal laws have been criticized for ignoring the irreducible specificity of biological contexts. Evolution does not always converge to optimal structures; it is shaped by historical contingencies, ecological constraints, and stochastic drift. Thus, the universality of evolutionary criticality or the directionality of the LIFI may be modulated by historical factors that break the idealization of global invariance [53].
(b) Incompleteness and Partial Unobservability: Concepts such as “elastic states” and “irreparable incompleteness” [20] suggest that it is not possible to derive universal axioms without incorporating the perspective of the system itself that enunciates them. Living systems possess degrees of self-reference—for example, cognition that models its own model—that hinder complete formalization. This imposes an intrinsic epistemological limit: the observer cannot be completely outside the system being studied.
(c) Ambiguity in the Definitions of Function and Information: Although the LIFI offers an attractive conceptual directionality, the objective measurement of functional information remains an open challenge. There are multiple metrics (effective information, Kolmogorov complexity, predictive capacity, structural redundancy), none of which fully captures the relationship between information and biological function. This methodological indefiniteness currently limits the predictive capacity of the law [33].

### 2.21. Falsifiable Predictions

(1) Fractal Scaling (WBE model): Metabolic rates and network geometries in extremophilic organisms, or in microbial ecosystems under high pressure, should conform to the scaling exponents predicted by the WBE model (where metabolic rate scales with body mass to the power of 3/4). Systematic deviations under conditions of energy boundaries—e.g., organisms living in extremely low energy conditions or with non-branching cellular architectures— would refute the universality of the model [13].
(2) Neuronal and Genetic Criticality: Cultured neural networks (brain organoids) or in vitro gene regulation systems should exhibit distributions of “avalanches” or expression fluctuations that follow power laws—a signature of self-organized criticality [10,14]. Genetic manipulations that alter connectivity (e.g., overexpression of synaptic channels or suppression of coupling proteins) should shift the system to subcritical or supercritical regimes, reducing information processing capacity and functional plasticity. This would offer a direct falsifiable test of the principle of functional criticality [54].
(3) Law of Increasing Functional Information (LIFI): Comparative paleogenomics allows testing of the LIFI hypothesis. If biological evolution follows a trend toward greater functional information, ancient genomes should show a systematic increase in effective information density, measurable by Kolmogorov complexity or integrated information in conserved genes [19]. A lack of net increase, or the presence of long-term reversible oscillations, would refute the directionality of the LIFI as a universal principle.
(4) Organizational Closure and Self-Fabrication: Synthetic biology offers an experimental avenue to evaluate organizational closure [55]. Protocellular systems designed with partially autocatalytic metabolic networks should show abrupt transitions to self-sustainment once a connectivity threshold is reached. If no such transition is observed—or if organizational closure requires external control—the universal applicability of the principle of autopoiesis and (M,R) systems would be called into question [56].

### 2.22. Conceptual Integration and Interdisciplinary Applications

The integrative framework of the universal organizational principles of biological systems transcends theoretical explanation to offer an operational language that connects physics, biology, and engineering. These principles—dissipation, organizational closure, fractality, modularity, criticality, degeneracy, antifragility, and increasing functional information—not only describe life [57]: they provide criteria for diagnosing, modeling, and designing living and artificial systems. Their value lies in the ability to translate theory into prediction and prediction into intervention.

### 2.23. Precision Medicine and Dynamic Pathophysiology

From this perspective, disease can be defined as a pathological phase transition, a deviation from the critical dynamic regime that characterizes healthy homeodynamics. Normal biological systems operate near the “edge of chaos,” where information processing is maximal and variability is functional; when the system shifts toward rigidity or disorder, pathological states emerge. Cancer can be reinterpreted as a breach of organizational closure: cells lose integration with the organism’s normative domain and acquire metabolic autonomy, breaking the system’s coherence [8]. Epilepsy represents a case of subcritical hypersynchronization: a loss of neural criticality that suppresses the dynamic diversity necessary for cognitive function [37]. Autoimmune diseases arise from a topological rupture of the self’s boundary, where the immune system confuses its own internal space with the environment. From this framework, precision medicine is redefined as a physics of living network control: therapies do not seek merely to inhibit molecules, but to restore critical dynamics and organizational closure. Therapeutic intervention becomes an engineering of the functional phase, aimed at redirecting the system’s dynamics toward viable attractors, assessable through metrics of entropy, criticality, and effective information processing [58].

### 2.24. Bioengineering, Synthetic Biology, and the Design of Life

Contemporary bioengineering faces the challenge of designing systems that not only function, but self-organize. The universal principles of biological organization provide a framework for this: hierarchical modularity and functional degeneracy offer design criteria for building robust and adaptive biological circuits, capable of maintaining functionality in the face of perturbations [9]. Fractal architecture inspires the design of tissues and microstructures that optimize transport, diffusion, and signaling, mimicking the geometries of vascular, neural, or lymphatic networks [10]. Synthetic autocatalytic and autopoietic systems offer a platform to explore the transition between complex chemistry and autonomous metabolism, experimentally testing the limits of organizational closure [12]. The synthetic biology of the future will be predictive if it adopts a thermodynamic and informational paradigm, designing organisms or ecosystems not by trial and error, but by following principles of dynamic stability, energetic coupling, and maximization of functional information.

### 2.25. Artificial Intelligence, Cognition, and Complex Systems

In the domain of cognitive sciences and artificial intelligence, the organizational principles of life offer a radically different model from the classic computational approach. Instead of networks trained on external data, life operates as a self-generating system of information, which actively learns by maintaining its internal coherence. The Free Energy Principle (FEP) [16] provides a unifying framework between biology and cognition: neurons, cells, and organisms all minimize surprise (prediction error) through active inference. From this perspective, biological cognition is redefined as a physical process of informational self-organization; biologically inspired artificial intelligence must be antifragile, that is, improve with perturbation and learn not only from data, but from errors and fluctuations; distributed AI systems—based on decentralized control, adaptive synchronization, and structural plasticity—represent the practical convergence between autopoiesis, criticality, and distributed cognition. This framework suggests a transition from “artificial intelligence” to organizational intelligence, where algorithms do not replicate the human mind, but the universal principles of life’s adaptation [59].

### 2.26. Towards a Technobiological Synthesis

The confluence of these fields points toward a new discipline: a predictive technobiology, where the physical principles of living organization guide both the understanding of biology and the creation of artificial systems that share its essential properties of autonomy, plasticity, degeneracy, and antifragility. In this context, the Physics of Life is not a return to mechanism, but the expansion of physics into domains where information acquires causal power. Its interdisciplinary application does not seek to control life, but to learn from its organizational grammar to design systems—medical, technological, or ecological—that share its capacity to persist, learn, and evolve [60].

## 3. Discussion

### 3.1. Critical Conclusions and Prospects for a Physics of Life

This review proposes that life is not an exception to physical laws, but a manifestation of them under conditions of flow, instability, and organizational closure. In this sense, the Physics of Life represents the most coherent contemporary attempt to formulate a unified framework that explains the emergence, persistence, and evolution of biological organization through general principles. From this effort, three fundamental theses emerge. Life as a Dissipative Structure with Organizational Closure: Organisms are materially open systems that maintain their identity through closed causal loops of production and regulation. Their teleonomy—the apparent purpose of living—is not a metaphysical attribute, but a physical consequence of the causal closure between metabolism, information, and boundary. Adaptation as a Critical and Antifragile Homeodynamics: Living systems self-regulate in a dynamic regime close to criticality, where stability and plasticity coexist. Functional degeneracy—multiple pathways for the same function—allows them to absorb perturbations and improve under stress, making antifragility a constitutive property of living. Cognition as Distributed Active Inference: Cognition emerges as a physical process of uncertainty minimization, extending from molecules to societies. It does not require a central controller; coherence arises from the synchronization and dynamic coupling between agents, formalized by the Free Energy Principle [61].

Thus, life emerges as a coherent, cyclic, and fractal expression of physical laws, where quantum electrodynamic coherence underlies organizational closure, energy storage, and functional degeneracy. This perspective not only unifies dissipation, criticality, and information, but also grounds biological teleology and cognition in the physics of coherent matter [29].

### 3.2. Epistemological Implications

The program of a “Physics of Life” requires abandoning the dichotomy between reductionism and holism. The challenge is not to derive biology from classical physics, but to expand physics to include systems where information and organization have causal power. This epistemological shift transforms the object of study from passive matter to organizing matter. Rather than seeking closed universal laws, the Physics of Life pursues principles of compatibility between scales, which explain how biological systems preserve their identity in fluctuating environments. This introduces a methodological change: laws are replaced by regimes of organization, defined by interaction topologies, information flows, and critical transitions.

### 3.3. Future Scientific Agenda

The development of this discipline will require coordinated efforts on three fronts. Multi-scale Formal Models: It is a priority to advance the unification of non-equilibrium thermodynamics, information theory, and network dynamics. Models that integrate organizational closure, energy flow, and active inference will allow the formulation of equations of state for living systems. Experimental Validation and Falsification: The experimental agenda must be directed at testing the falsifiable predictions derived from the theoretical framework: the universality of criticality in organoids, the increase in functional information in evolutionary lineages, and the emergence of autocatalytic closure in synthetic systems. Technobiological Applications: The future of bioengineering and artificial intelligence will depend on incorporating these principles into the design of systems that learn, adapt, and evolve on their own. The engineering of the 21st century will not only build machines that work, but systems that live.

### 3.4. Towards a Physics of Creative Order

The Physics of Life is emerging as the science of creative order: it is the study of how matter, by dissipating energy, generates organization, meaning, and operational purpose. Its consolidation will mark a new phase in the history of knowledge, a truly integrative science where life, mind, and evolution are understood as expressions of the same organizational grammar. The challenge is not minor: to understand how nature turns disorder into meaning. But in that quest lies the promise of a theory capable of reconciling physics with biology—and, ultimately, with the very experience of being alive. The future lies not only in finding more universal laws, but in understanding the principles by which life navigates uncertainty and creates its own order.

### 3.5. Limitations of the Study

While this theoretical framework offers a unifying perspective on biological organization, several limitations should be acknowledged.

Theoretical and Conceptual Limitations: The proposed universal principles, while compelling, remain largely theoretical and require more extensive empirical validation across diverse biological systems. The framework’s broad scope necessarily sacrifices some biological specificity, potentially oversimplifying the intricate details of particular organisms or evolutionary contexts.

Measurement Challenges: Quantitative assessment of key concepts such as “functional information,” “organizational closure,” and “criticality” in real biological systems presents significant methodological challenges. Current metrics may not fully capture the multidimensional nature of these properties across different scales of biological organization.

Historical Contingency vs. Universal Principles: The tension between universal physical principles and historical evolutionary contingency remains unresolved. While the framework emphasizes universal organizational patterns, it may underweight the role of stochastic events, path dependence, and unique historical circumstances in shaping biological systems.

Scale Integration Challenges: Although the holographic principle suggests self-similar organization across scales, the specific mechanisms translating organizational principles from molecular to ecosystem levels require more detailed elaboration. The framework currently lacks comprehensive mathematical formalisms bridging these scale transitions.

Experimental Validation Gaps: Many predictions derived from this framework, particularly those concerning evolutionary directionality and critical transitions, require long-term experimental studies that are challenging to implement. The falsifiability of some principles remains limited by current technological capabilities.

Computational and Modeling Limitations: The complex, non-linear dynamics described in this framework pose significant challenges for computational modeling and simulation. The current modeling approaches may be insufficient to capture the full richness of the multi-scale biological organization described by these principles.

These limitations highlight the need for continued interdisciplinary research to refine, test, and potentially revise the proposed framework through the integration of theoretical insights with empirical data from diverse biological systems.

## 4. Conclusions

Life can be interpreted as a regime of organized matter sustained far from thermodynamic equilibrium, in which energy dissipation, organizational closure, multiscale architecture, and information processing become dynamically coupled. From this perspective, the Physics of Life is not a metaphorical extension of physics to biology, but an attempt to identify the physical constraints and organizational principles under which living systems emerge, persist, adapt, and evolve.

The synthesis developed here suggests that dissipation alone is not sufficient to explain life. Biological organization requires the coordinated integration of non-equilibrium thermodynamics, self-fabrication, functional closure, critical dynamics, and distributed inference-like processes. In that sense, living systems are not merely structures that resist disorder; they are systems that stabilize themselves by exploiting flows of energy and information to maintain coherence, regulate internal states, and generate adaptive responses across scales.

This framework supports three general conclusions. First, biological order is a dynamic achievement rather than a static condition: life persists through continuous turnover, regulation, and reorganization under thermodynamic constraints. Second, autonomy is physically grounded in organizational closure, whereby systems actively produce and preserve the components and relations that sustain their own viability. Third, cognition should be understood in an expanded sense, as a distributed process of state estimation, error correction, and adaptive action that links metabolism, regulation, and behavior rather than as an exclusively neural property.

The integrative value of this framework lies in its capacity to generate testable consequences. Its scientific relevance will depend on whether principles such as criticality, fractal scaling, organizational closure, and increasing functional information can be operationalized, measured, and falsified across diverse biological systems. Thus, the next stage is not rhetorical synthesis but empirical discrimination: identifying which proposed principles correspond to robust organizational constraints, which are scale-dependent regularities, and which remain only suggestive analogies.

Future work should therefore advance along three fronts: the development of formal multiscale models linking thermodynamics, network organization, and information processing; the experimental testing of predicted regimes in organoids, microbial systems, immune networks, and synthetic protocellular platforms; and the translation of these principles into precision medicine, synthetic biology, and biologically inspired artificial intelligence.

Taken together, these considerations support a restrained but substantive conclusion: life, mind, and evolution may be understood as different expressions of the same underlying problem—how matter, under physical constraint, becomes capable of maintaining organization, producing functional coherence, and adapting through time. If this view is correct, the Physics of Life will matter not because it offers a final theory of biology, but because it provides a rigorous framework for asking which forms of biological order are possible, which are stable, and which are inevitably constrained by the physics of organized matter.

## Data Availability

This paper analyzes existing, publicly available data. All data and theoretical models discussed are available from the cited references. This paper does not report original code. Any additional information required to reanalyze the data reported in this paper is available from the corresponding author upon request. This study did not generate new unique reagents.
